# Magnetic Application of Gadolinium Orthoferrite Nanoparticles Synthesized by Sol–Gel Auto-Combustion Method

**DOI:** 10.3390/gels8110688

**Published:** 2022-10-25

**Authors:** Loganathan Guganathan, Chinnaiyan Rajeevgandhi, Kaliyamurthy Sathiyamurthy, Kokila Thirupathi, Madhappan Santhamoorthy, Ellappan Chinnasamy, Chaitany Jayprakash Raorane, Vinit Raj, Seong-Cheol Kim, Pichapillai Anand

**Affiliations:** 1 Department of Physics, Annmalai University, Annamalainagar, Chidambaram 608002, Tamilnadu, India; 2 Freelance Researcher, Chidambaram 608001, Tamilnadu, India; 3 Department of Physics, Sri Indu College of Engineering & Technology, Ibrahimpatan 501510, Telangana, India; 4 Department of Physics, Sri Moogambigai College of Arts and Science for Women, Palacode 636808, Tamilnadu, India; 5 School of Chemical Engineering, Yeungnam University, Gyeongsan 38544, Korea

**Keywords:** GdFeO_3_, Sol–Gel auto-combustion method, synthesis, characterization

## Abstract

In this manuscript, we present the synthesis of gadolinium orthoferrite nanoparticles using the sol–gel auto-combustion technique. The obtained gadolinium orthoferrite nanoparticles were annealed at various temperatures, such as 800 °C, 900 °C, 1000 °C, and 1100 °C. The synthesized materials were analyzed by various instrumental characterizations. The vibrational characteristics of the synthesized samples were verified by FTIR. The surface morphology of the gadolinium orthoferrite nanoparticles was analyzed by FE-SEM and HR-TEM, revealing their spherical structural morphology and uniform particle structure. The presence of the elemental features was analyzed in the gadolinium orthoferrite nanoparticles by EDAX. The surface analysis of the core ranges of the XPS-recorded spectra were obtained for the elemental states of the Gd, Fe, and O factors in the samples, and it additionally characterized the different levels of oxidative states by fitting the levels of the high-resolution parameters of Gd 4d, Fe 2p, and O 1s. The magnetic properties of the samples were investigated by VSM. The measurement of the magnetic parameters revealed that gadolinium orthoferrite nanoparticles exhibit a ferromagnetic nature.

## 1. Introduction

Perovskite-type materials have been emerging in importance in recent years due to their extensive applications in different technologies. Perovskite materials’ photochromic behavior might open up applications in consumer products and electronic devices. There is visible photoluminescence peak in lead-halide perovskite materials under a two-photon absorption process while tuning the excitation wavelength. Recently, several lead-halide perovskites have been demonstrated to be functional materials with hydrochronic, thermochromic, and photochromic properties for anticounterfeiting applications [1,2,3]. GdFeO_3_ is one of the most vital types of lanthanide metal-oxide perovskites ABO_3_ (A = La, Sm, Eu, Gd). The best perovskite has a cubic crystal structure, composed of a 3D shape of corner-sharing BO_6_ octahedral B-sites [4]. Orthoferrite materials have more properties, including optical, electrical, and magnetic ones; therefore, they are technologically and scientifically significant [5]. RE orthoferrites’ (ReFeO_3_) crystals have an orthorhombic, distorted perovskite-type belonging to Pbnm, with the space group D162h. ReFeO_3_ materials exhibit interesting physical and chemical properties due to their ionic and electronic defects [6]. The ionic radius of Gd^3+^ is 0.093 nm, which is superior to that of Fe^3+^, which is 0.067 nm, and as a result, the quantity of Fe^3+^ ions replaced by Gd^3+^ ions is confined, and solubility restriction also occur in the replacement of Fe^3+^ ions by Gd^3+^ ions. For this reason, it is expected that an excess substitution of Gd^3+^ ions will tend to aggregate around the grain boundaries in the form of GdFeO_3_ [7]. Gadolinium orthoferrite nanoparticles have been successfully obtained by various synthesis methods, including co-precipitation [8], sol–gel [9,10,11,12], and microwave [13,14,15]. As compared to other materials, gadolinium orthoferrite nanoparticles are generally considered as efficient materials in various applications, including better gas-sensing performance and medical applications, and gadolinium complexes are the most widely used of all MRI contrast agents [16]. The present work focuses on the synthesis of perovskite GdFeO_3_ nanoparticles using the sol–gel auto-combustion technique. The emergence of this material with a controlled size and shape is scientifically necessary due to the strong correlation between these parameters and their magnetic properties. Herein, we discuss how GdFeO_3_ nanoparticles can be successfully prepared on a large scale via the sol–gel technique. The size and shape of the final product it can be readily tuned in the extensive range by tuning-process likewise simple techniques, low cost, high yield, an eco-friendly, the reaction temperature time and molar ratio. Our special observation is made based on the characterization of the thermal, structural, and magnetic behavior of the given material, with great importance in the role of its structure and enhanced magnetic properties.

## 2. Results and Discussion

### 2.1. Thermal Analysis

**Figure 1:**
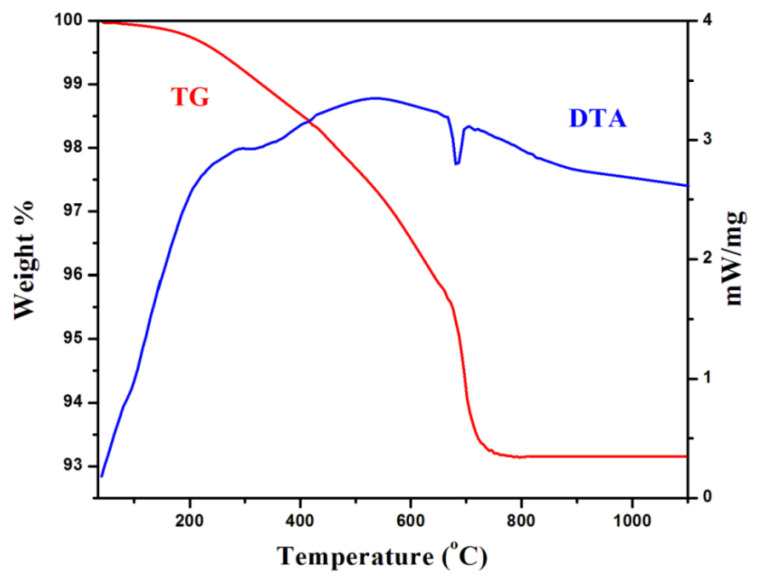
TG-DTA pattern of as-prepared gadolinium orthoferrite.

The TG-DTA of the prepared GdFeO_3_ nanoparticles, which were measured in the temperature range of 35 °C to 1100 °C at the rate of 20 °C/min, is shown Figure 1. In this plot of the TGA curve, three predictable weight losses were measured between in range between 35 °C and 1100 °C. The absorption of water molecules was observed at 180 °C during the first weight loss. The absorption of organic templates was observed in the ranges 180 °C to 410 °Cand measured in second weight loss. The crystallization of the final product in the range between 410 °C and 650 °C was due to the third weight loss. No evidential weight loss was identified beyond the 720 °C range, which indicates the formation of GdFeO_3_ nanoparticles. A broad endothermic peak at 300 °C was exhibited in the DTA curve, owing to the dehydration. Three stages of the decomposition of the anhydrous precursor were observed after dehydration. In conclusion, the as-prepared perovskite gadolinium orthoferrite nanoparticle revealed the phase transition of the materials beyond 720 °C [17].

### 2.2. XRD Analysis

**Figure 2:**
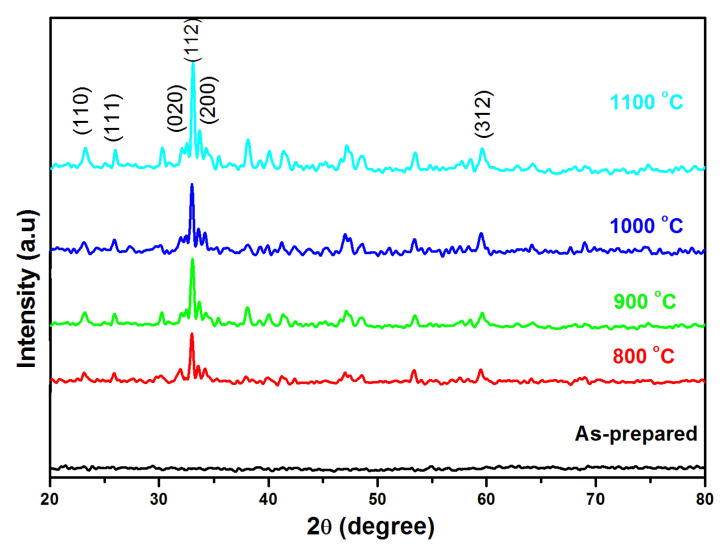
X-ray diffraction of gadolinium orthoferrite annealed at 800 °C, 900 °C, 1000 °C, and 1100 °C.

The XRD features of the as-synthesized material and the different levels at 800 °C, 900 °C, 1000 °C, and 1100 °C of the annealed GdFeO_3_ nanoparticles are shown in Figure 2. As can be seen from the as-synthesized product, no crystallographic peaks appeared. This indicates that the annealing process is crucial to steadying the crystallites’ sizes and to avoiding their agglomeration. The increase in the annealing temperature was mostly correlated with variations in the material microstructure during the annealing and the thermally induced ordering or reordering of the material. The XRD pattern of all of the annealed gadolinium ferrites exhibits peaks with h, k, and l values of (110), (111), (020), (112), (200), and (312), and the planes indicate the perovskite-like orthorhombic type. The crystallographic peaks are well-matched with JCPDS card # 78-0451. The clarity of the observed peaks shows the formation of gadolinium orthoferrite nanoparticles. The mean crystallite sizes were calculated using the Debye-scherrer’s formula [18].

Table 1 shows that the increasing temperature levels of the annealing process result in the gradual increase of the crystallite size. The crystallite size increases are 20, 23, 25, and 27 nm with respect to the increasing temperatures of 800 °C, 900 °C, 1000 °C, and 1100 °C. The observed behavior revealed the development of the gadolinium orthoferrite nanoparticles. We have analyzed the prepared GdFeO_3_ nanoparticles using XRD analysis to characterize the primary confirmation of the formed GdFeO_3_ crystal structures. We found that the materials that were annealed at 800 °C showed better crystallinity order than did the other samples, which were annealed at 900 °C and 1000 °C. Therefore, we chose to use the sample prepared at 800 °C for all of the other characterization techniques.

### 2.3. Infrared Spectroscopy

**Figure 3:**
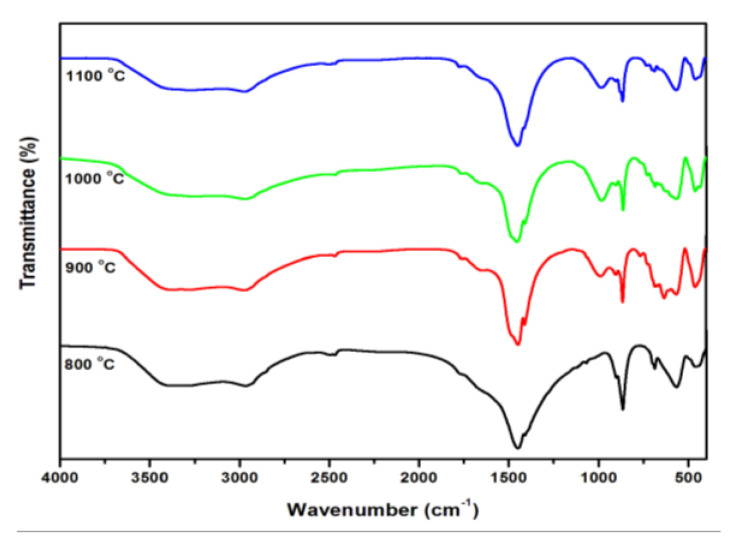
FTIR spectra of GdFeO_3_ nanoparticles annealed at temperatures of 800 °C, 900 °C, 1000 °C, and 1100 °C.

The FTIR spectra of the GdFeO_3_ nanoparticles performed in the range of 4000–400 cm^−1^ is shown in Figure 3. It is clear from the FTIR analysis of the different levels of the annealed GdFeO_3_ samples that, in the lower-wave numbers of the region, strong absorption bands are around 559–561 cm^−1^. The observed values are assigned as GdO and FeO due to the formation of stretching vibrations in Gd-O-Fe and Fe-O-Fe [19]. In the higher wave number region, two vibrations are observed at 554–556 and 593–597 cm^−1^. As a product of evaluation with the wave numbers of vibrations of natural cubic iron and Gd-O, it can be assumed that these two vibrations are most likely related to the Gd-O and Fe-O stretching vibrations, respectively. The observed values of the absorption bands around 432–436 cm^−1^ are most likely credited to the O-Fe-O bending vibrations in the octahedral B-site, respectively [20,21]. We concluded that the recorded spectra confirm the absorption bands between 441–443, 554–556, and 593–597 cm^−1^ are characteristic of gadolinium orthoferrite. This observation reveals some shift in the vibrations in the FTIR spectra of the annealing samples in contrast with the sol–gel hydroxides; this may be due to effect of the formation of the α-GdFeO_3_ crystal structure.

### 2.4. FE-SEM with EDAX Analysis

**Figure 4:**
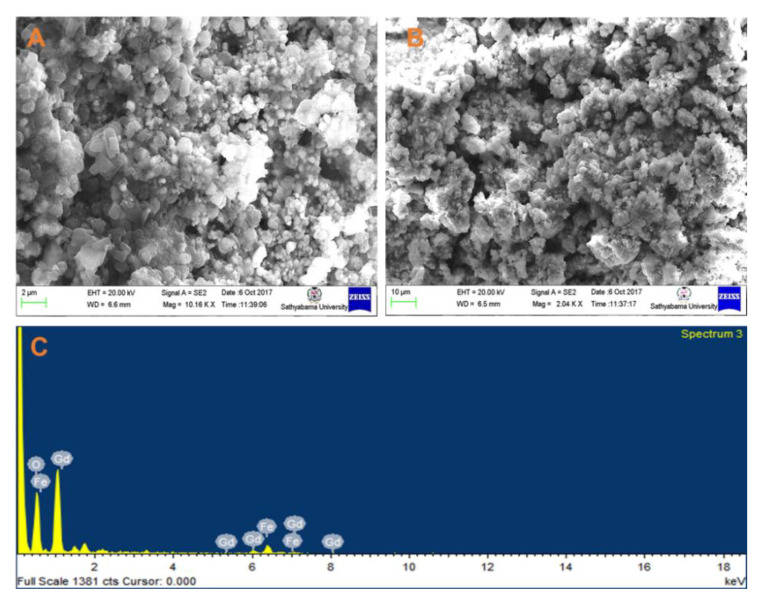
FE-SEM image of GdFeO_3_ nanoparticles annealed at a temperature of 800 °C (**A**,**B**) and the corresponding EDAX mapping (**C**).

The FE-SEM images of the gadolinium orthoferrite nanoparticles annealed at 800 °C are shown in Figure 4. It is clear from the micrograph that gadolinium orthoferrite exhibits aspherical type with some agglomeration. The agglomeration of the prepared gadolinium orthoferrite nanoparticles indicates the magnetic interaction between the samples. The elemental composition of GdFeO_3_ was investigated with EDX, and it contained Fe, Gd, and O. From the Energy Dispersive X-ray Analysis data, the Gd, Fe ratio of the samples was 1:2. No additional lines were detected in the elemental mapping, confirming that they were pure gadolinium orthoferrite nanoparticles.

### 2.5. HRTEM Analysis with SAED

**Figure 5:**
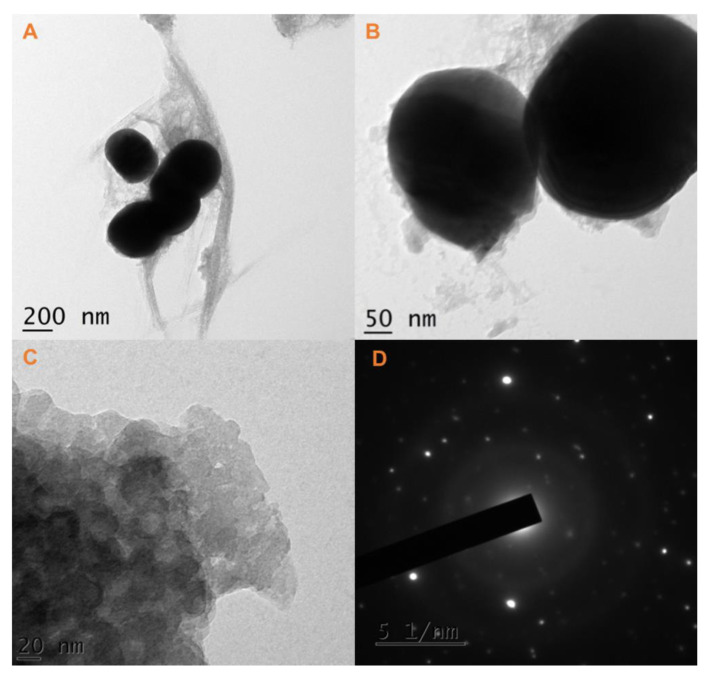
HRTEM images of GdFeO_3_ nanoparticles annealed at a temperature of 800 °C (**A**–**C**) and the corresponding SAED pattern for Figure 5c (**D**).

An HRTEM micrograph of the gadolinium orthoferrite nanoparticles annealed at 800 °C is shown in Figure 5. In this work, perovskite and orthorhombic-type gadolinium orthoferrite nanoparticles were present with a spherical structure [22]. The spherical structure was measured using ImageJ viewer software, demonstrating that the perovskite-like orthorhombic type of gadolinium orthoferrite nanoparticles’ size was 15 nm. In addition, the SAED pattern of the perovskite-like orthorhombic structure for the gadolinium orthoferrite nanoparticles revealed that they joined to diverse diffraction planes. The clear diffraction spots disclose good dispersion and good crystallinity behavior.

### 2.6. X-ray Photoelectron Spectra

**Figure 6:**
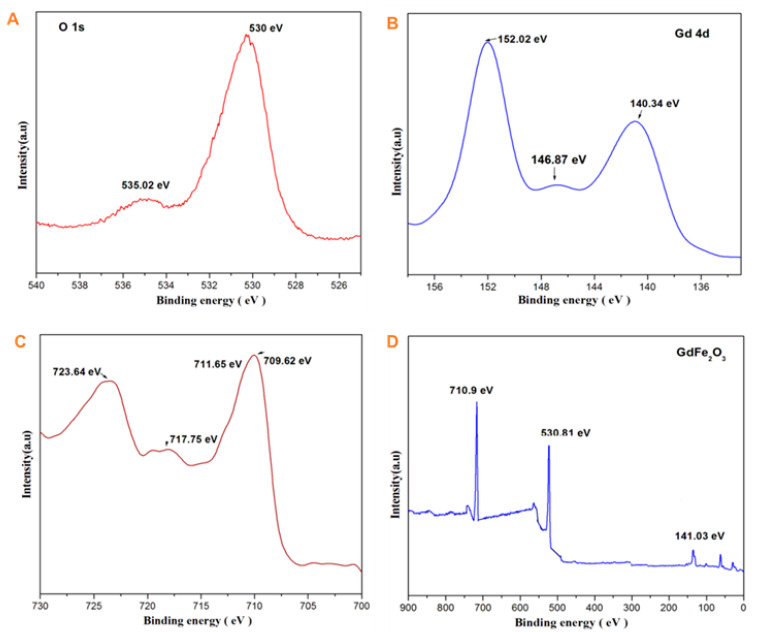
(**A**–**D**) XPS spectra of GdFeO_3_ nanoparticles annealed at a temperature of 800 °C.

Figure 6A–D shows the oxidation states of the metals in the GdFeO_3_ sample as examined by X-ray photoelectron spectroscopy. The XPS spectra of the GdFeO_3_ powder annealed at 800 °C reveals the presence of Gd, Fe, and O. Therefore, the equivalent XPS peaks were used to find the relative contribution of the distinct oxidation state [23]. As shown in Figure 6A, the two prominent peaks at 530 eV and 535.02 eV indicate the O1s. Figure 6C the observed peaks around 709.62, 711.65, 717.75, and 723.64 are preferred to the Fe^3+^ at the octahedral and tetrahedral sites, respectively. In addition, a satellite peak was noticeable at a binding energy of around 709.62 eV. On the basis of the spin-orbit coupling rule, the Fe 2p orbital exhibits a doublet of Fe 2p3/2 and Fe 2p1/2 peaks at binding energies of 709.62 and 723.96 eV, attributed to the exchange interaction between the outer 3d electrons and the remaining 3s electron of the atom. We may conclude from Figure 6B that the XPS analysis for Gd 4p and Gd 4d regions of gadolinium show that they are incorporated in ferrite. The spectra of the prepared samples contain foremost binding energy peaks present around 152.02, 146.88, and 140.34 eV, respectively. From the graph, we can see the presence of a shoulder peak at 146.88, 140.34 eV (major) assigned to Gd^3+^. Additionally, a satellite peak was observed at 152.02 (minor). The major O 1s peak observed at 530 for the prepared samples correspond to O^2−^ anions in the gadolinium orthoferrite crystal lattice. The second peak that appeared at 535.02 eV could be recognized as under-coordinated lattice oxygen, recommending a structural defect, which is shown in Figure 6A. The measured gadolinium orthoferrite oxidation state values are presented in Table 2. From the XPS analysis, it may be concluded that the prepared gadolinium orthoferrite nanoparticles survived in multiple oxidation states [24].

### 2.7. Magnetic Analysis

**Figure 7:**
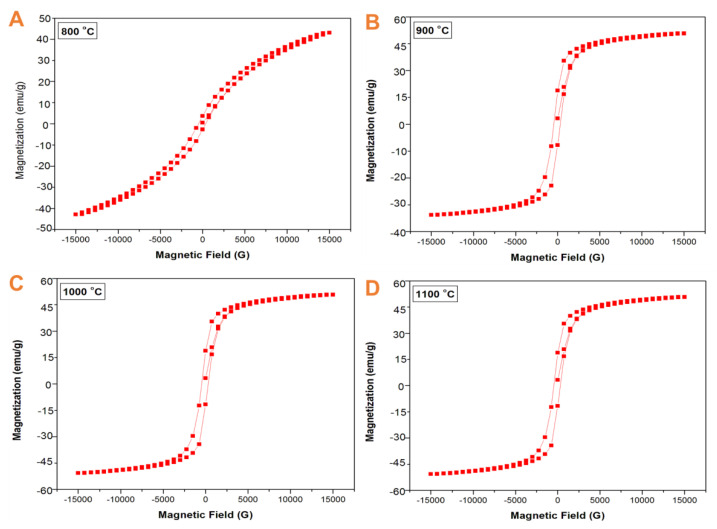
(**A**–**D**)**.** Magnetization curves of the GdFeO_3_ nanoparticles at different temperatures.

Magnetic measurements of the GdFeO_3_ nanoparticles at various annealing temperatures were recorded as falling between −5G and +15G as performed by VSM. Figure 7 depicts the room-temperature magnetic hysteresis curve of the GdFeO_3_ nanoparticles annealed at the different temperatures of 800 °C, 900 °C, 1000 °C, and 1100 °C. The measurement of the saturation magnetization (Ms), and Coercivity (HC) values are discussed. As shown in Table 3, when the annealing temperature increased, the related saturation magnetization (Ms) value increased from 33.3 emu/g to 53 emu/g, and the Coercivity (HC) value increased from 544 G to 729 G. The Coercivity (Hc) is the magnetic field necessary for overcoming the magneto-crystalline anisotropy to turn over the magnetic momentum. In the GdFeO_3_ perovskite structure, Fe^3+^ ions are bounded by six O^2−^ ions in an octahedral symmetry; as an effect, the Fe^3+^ ions interact with O^2−^; therefore, a maximum number of unpaired electrons are generated, which results in magnetic momentum. In GdFeO_3_, both the Fe^3+^ and Gd^3+^ ions have magnetic properties, and the resultant magnetic momentum comes from the donation of both the ions [25]. In the present work, the different levels of annealed samples confirmed the existence of a ferromagnetic nature. As observed in Figure 7A–D, the magnetization curve for the sample prepared at 800 °C showed a different curve pattern than those of the other samples, which were prepared at 900 °C, 1000 °C, and 1100 °C, respectively. We believe that this might be caused due to the change of the order of magnetization properties of the GdFeO_3_ nanoparticles from a weak ferromagnetic arrangement to a strong ferromagnetic order, with respect to the increase in the annealing temperature.

## 3. Conclusions

In the present work, GdFeO_3_ nanoparticles were synthesized using the sol–gel auto-combustion technique, and the structural, morphological, and magnetic properties of GdFeO_3_ depend on several factors, such as the experimental synthesis, chemical composition, and particle size. The formation of orthorhombic ferrite was confirmed by XRD spectra, and the crystallite sizes were found to increase with the increase in the annealing temperature. FTIR analysis revealed the formation of a ferrite structure with two strong peaks at around 575–583 cm^−1^ and 432–438 cm^−1^, respectively. The HRTEM results revealed perovskite and orthorhombic-type gadolinium orthoferrite nanoparticles present in a spherical structure, and the particle size was 15 nm. XPS analysis confirmed that the gadolinium orthoferrite nanoparticles survived multiple oxidations. VSM analysis revealed that the different levels of annealed gadolinium orthoferrite samples increased with increases in saturation magnetization (Ms) from 33.3 to 53 emu/g, and the Coercivity (HC) value was 544–729 G, which confirmed the existence of a ferromagnetic nature. The prepared material is promising for magnetic recording and applications.

## 4. Materials and Methods

For the synthesis of gadolinium orthoferrite using the sol–gel auto-combustion technique, 0.2 M of ferric nitrate, 0.1 M gadolinium nitrate, and 0.29 M of citric acid were dissolved in 20 mL of de-ionized water individually, and those solutions were added one by one, respectively. A suitable amount of ammonia solution was added to maintain the pH level. The prepared solution was magnetically stirred for 5 h at 70 °C. A black-colored gel appeared, and after few minutes, it burned via auto-combustion, and the residual product was obtained. Afterwards, the burned powder was dried in a hot-air oven for 2 h at 100 °C. The forms of gadolinium orthoferrite powder samples were annealed 2 h at different temperature levels, such as 800 °C, 900 °C, 1000 °C, and 1100 °C. Furthermore, the different levels of annealed powder were characterized by XRD, FTIR, FE-SEM with EDAX, HRTEM with SAED, XPS, and VSM.

In this work, we chose the sol–gel auto-combustion method to prepare GdFeO_3_ nanoparticles due to their advantages, such as the strong correlation between these synthetic parameters, including large-scale production, reaction time, and combustion temperature, which control the magnetic properties of GdFeO_3_ materials. The size and shape of the final products can be readily tuned in a wide range by tuning process parameters, such as a simple method, a high yield, being eco-friendly, the reaction temperature, and the molar ratio of the material.

### Characterization Techniques

The synthesized gadolinium orthoferrite powders were characterized by employing different characterizations. A TG-DTA study was performed at a heating rate of 10 °C min^−1^ in an air atmosphere using a NETZSCH-STA 449 F3 JUPITER. The phase confirmations of the annealed gadolinium orthoferrite were performed using an X-ray diffraction (XRD)PANalytical XPERT-PRO with monochromatic Cu Kα radiation (λ = 1.54060 Ǻ) at 30 mA and 40 kV with a 0.05 step in the 2θ range of 20°–80°. FTIR spectra were carried out using a Perkin Elmer Spectrum BX model infrared spectrophotometer recording in the range from 4000–400 cm^−1^. Structural measurements were carried out using a field-emission scanning electron microscope (SUPRA 55, CARL ZEISS, GERMANY) to record the FE-SEM with EDAX. Ahigh-resolution transmission electron microscope (HRTEM) Jeol/JEM 2100, with a source of LaB6 and with a resolution 0.23nm, a lattice of 0.14 nm, with a voltage of 200 kV was used. XPS studies were performed using a Thermo Fisher Scientific ESCALAB 250Xi X-ray spectrometer. The magnetizations of GdFeO_3_ were measured with a vibrating sample magnetometer, instrument model 7407, Lakeshore, USA, with a maximum magnetic field of 2.5 T, with a dynamic moment range of 1 × 10^−6^–1 × 10^3^ emu M-H at room temperature. The magnetic measurement of the gadolinium orthoferrite was carried out by VSM.

## Figures and Tables

**Table 1:** Crystallite sizes of the gadolinium orthoferrite.

GdFeO_3_	Crystallite Size (nm)
800 °C	20.2
900 °C	23.6
1000 °C	25.1
1100 °C	27

**Table 2:** XPS spectra of gadolinium orthoferrite annealed at 800 °C.

Element	Peak Binding Energy
Gd 4d	152.02 146.87 140.34
Fe 2p	709.62 717.75 723.64 711.65
O 1s	530 535.02

**Table 3:** Magnetization of gadolinium orthoferrite.

Temperature	Saturation Magnetization (Ms) emu/g	Coercivity G
800 °C	33.1	544
900 °C	38.7	605
1000 °C	47.2	684
1100 °C	53.1	729

## Data Availability

Not applicable.
